# The compressive strength development and pH of cement mortars incorporating high volume supplementary cementitious materials under accelerated curing

**DOI:** 10.1016/j.heliyon.2025.e42240

**Published:** 2025-01-24

**Authors:** Sumra Yousuf, Muhammad Rizwan, Belal Alsubari, Mustabshirha Gul, Muhammad Mahmood Ali, Muhammad Nasir Bashir, Abid Latif

**Affiliations:** a Department of Building and Architectural Engineering, Faculty of Engineering & Technology, Bahauddin Zakariya University, 60000, Multan, Pakistan; b Metallurgical Engineering Department, NED University of Engineering and Technology, 74200, Karachi, Pakistan; c Department of Civil Engineering, Faculty of Engineering, Miami College of Henan University, Kaifeng, Henan, China; d Department of Mechanical Engineering, Faculty of Engineering & Technology, Bahauddin Zakariya University, 60000, Multan, Pakistan; e Department of Mechatronic Engineering, Atlantic Technological University Sligo, Ash Lane, Sligo, F91 YW50, Ireland; f Multi-Scale Fluid Dynamics Lab, Department of Mechanical Engineering, Yonsei University, Seoul, 120-749, Republic of Korea; g Department of Civil Engineering, Faculty of Engineering & Technology, Bahauddin Zakariya University, 60000, Multan, Pakistan

**Keywords:** pH value, Supplementary cementitious materials, Mortar, Curing, Ca(OH)₂

## Abstract

The durability and service life of concrete structures is primarily dependent on the performance of cement-based material (CBM). This performance of CBMs is linked to their pH directly. Literature suggests that when supplementary cementitious materials (SCMs) are used, the pH of CBMs decreases as a result Ca(OH)₂ consumption during the Pozzolanic reaction (PR). With the construction industry increasingly adopting blended cements for their technical, economic, and environmental benefits, the use of high volumes of SCMs remains a subject of caution. This study investigates the pH behavior of cement mortars modified by replacement of ordinary Portland cement (OPC) with three SCMs (50 %): fly ash (FA), ground granulated blast furnace slag (GGBS), and treated palm oil fuel ash (TPFA), both at early and later stages of curing. The pH values were measured at different curing ages, and thermal gravimetric analysis and X-ray diffraction were used to support the findings. Results indicated that the pH of mortars containing FA or GGBS showed only a slight decrease after 5 months compared to their initial pH values, remaining above 11.5, which is considered within the safe range for concrete durability. In contrast, the pH of TPFA blended mortars decreased significantly, dropping by approximately 14 %, indicating that TPFA had a more substantial impact on the alkalinity of the mortars. These findings suggest that the pH of CBMs is influenced by factors other than just the Ca(OH)₂ (Hydrated lime) content, such as the chemical composition and reactivity of the SCMs used, and that high volumes of TPFA may negatively affect long-term durability.

## 1 Introduction

The pH behaviour of CBMs primarily drives the most significant features of the concrete structures, such as; durability and service life [1,2]. The CBMs including paste, mortar and concrete, begin their cycle owing high pH (approximately 12.5–13.5) [3,4].

The portlandite Hydrated lime along with alkali metals are responsible for high pH of CBMs [3,5]. On the other hand, high pH of CBMs may be controlled by consumption of portlandite in the formation of hydration products and/or carbonation. This pH reduction will diminish the passivation layer of concrete reinforced cements [6].

Numerous factors including chloride ingress, acid attack, carbonation and biodegradation will reduce the pH of the concrete. Low pH will diminish the passive protective layer of the embedded steel bar in concrete, and increase the risk of rebar corrosion, which will lead to problems including chipping, spalling and cracking of concrete cover. The pH of CBMs can be reduced by utilizing SCM which consumes Hydrated lime during PR [7]. When SCMs are added, the total cement content reduces, which lead to less formation of Hydrated lime during hydration of the cement [8]. The utilization of high volumes of SCMs as cement replacement, such as fly ash (FA) from 25 to 30 % [9], GGBS from 25 to 65 % [10] and PFA from 10 to 50 % [11] in CBMs might consume most of the Hydrated lime in the PR, and destroy the pH buffer capacity of the concrete.

In various constructional works, such as load-bearing walls, beams and columns, the optimal quantity of FA for the replacement of cement is approximately 15%–25 % [12,13]. In massive structures, such as foundations, footings and dams, 30%–50 % FA can be used [14]. The studies have reported that 40%–60 % FA can be utilised to replace cement in structural applications and to produce concrete with good mechanical properties [15]. Durability issues related to the carbonation and cracking of concrete, low early-age strength, slow strength development and prolonged setting time can be addressed by using high amounts of FA in CBMs [16].

In CBMs, the optimal quantity of GGBS in the replacement of cement is approximately 50 % [17,18]. The long-term properties of CBMs incorporating GGBS can be improved for all types of structural construction works, such as foundations and marine concrete structures. However, various problems, such as low strength and poor durability at early ages, may be encountered due to the substitution of cement with high levels of GGBS in CBMs [19,20].

Several research reports [[21], [22], [23]] state that the replacement level of ground PFA (G-PFA) should not exceed 20%–30 %. In CBMs, high G-PFA substitution levels increase the demand of water and simultaneously reduce the early-age compressive strength (CS). However, the shortcomings arising from the use of G-PFA can be addressed via heat treatment (HT) and regrinding after HT in order to yield particles with improved pozzolanic effect and increased fineness. This newly developed PFA is called treated PFA (TPFA), which can be used at the cement replacement level of up to 50%–70 % in CBMs [[24], [25], [26]].

The industry is using one or more types of SCMs in concrete instead of only pure cement [[27], [28], [29]] but remains hesitant about using high fractions of SCMs. One of the causes of this hesitation is the durability problems induced by the consumption of Hydrated lime and dropping in the concrete's pH. pH is one of the important factors determining the durability of concrete structures. However, limited information regarding the pH behaviour of high-volume CBMs is available in literature. Therefore, the primary objective of this study is to evaluate the short and long-term changes in pH values of mortars having 50 % SCMs when cured under normal or accelerated curing patterns. X-ray diffraction analysis (XRD) and thermal gravimetric analysis (TGA) were carried out to support the findings.

## 2 Experimental settings

### 2.1 Materials used

Ordinary Portland cement (OPC) with a specific gravity (SG) of 3.1 and specific surface area (SSA) of 2667.24 cm^2^/gm was used in all mixes. The whitish grey class F Fly Ash with a SG and SSA of about 2.29 and 2858.6 cm^2^/gm, and GGBS with 2.83 and 3197.2 cm^2^/gm respectively were used as common cementitious materials.

The original and raw PFA was taken from a palm oil mill in the Selangor state of Malaysia, which was treated to yield TPFA. The drying of raw PFA was performed in an oven for a period of 24 h at 105 °C with a tolerance of 5 °C. The sieving (30 cm diameter) was employed to separate the uneven residues of PFA. In next phase, Los Angeles abrasion equipment was used to yield smaller particle size. The (7 Kg) PFA was ground at 33 rpm for 16 h. Later PFA's loss on ignition (LOI) was reduced by holding at 600 °C for 120 min. Further grinding was carried out in order to enhance fineness. The SG of TPFA was found to be 1.98 while its SSA was calculated to be 7400 m^2^/kg.

The fine aggregate owing SG 2.68 and sand grain size's maximum limit of 4.75 mm was used for this study. Solution of modified polycarboxylate copolymers with the density of 1.09 kg/m^3^ was utilised as the superplasticiser (SP) to obtain cement mortars with good workability. Normal tap water was used for mixing and curing of specimens. Chemical compositions of powders were determined via the X-ray fluorescence spectrometry test. LOI was determined by heating the samples at 900 °C-1000 °C. The results are presented in Table 1. As shown in the table, the MgO content of the FA used in this study exceeded 6 %. The ASTM C150 [30] and standards in some countries [31] state that the MgO content of cement should be limited to 6 %. Although the MgO content of FA has no limits in this standard, it was specified in the BS EN 450–1:2005 [32] that it should not exceed 4 % (by mass). Therefore, the effect of using FA with high MgO content on the volumetric instability of concrete materials should be further investigated.

**Table 1:** Oxide compositions (% by mass) and LOI values of OPC, FA, GGBS and TPFA.

Compound name	OPC	FA	GGBS	TPFA
SiO_2_	20.14	39.86	36.01	69.02
CaO	60.82	12.72	40.54	5.01
Al_2_O_3_	3.89	17.10	13.17	3.9
MgO	3.10	6.79	5.42	5.18
Fe_2_O_3_	3.35	14.98	0.42	4.33
P_2_O_5_	0.064	0.20	0.011	–
MnO	0.14	0.18	0.18	–
K_2_O	0.24	1.03	0.34	6.9
TiO_2_	0.16	0.89	0.57	–
SO_3_	2.25	0.58	1.77	0.41
SrO	0.02	0.06	0.05	–
LOI	2.3	0.70	0.72	2.1

### 2.2 Mortar mixtures and curing patterns

As can be seen in Table 2, four different mortar mixtures were studied. The control mortar was designed with a cement:sand ratio of 1:3 and the water:binder ratio of 0.48. In blended cement mortars, 50 % of the cement was replaced with FA, GGBS and TPFA by weight. Additional water and SP were used to obtain a Cement-TPFA mortar mix with the same workability as all other mortar mixes. This mortar mix had reduced workability because TPFA contained unburned carbon that absorbed additional water and SP [33,34].

**Table 2:** Batch wise composition of mortar mixes.

Mix	Binder (kg)				Water (kg)	w/b	Sand (kg)	SP (% of binder)
	OPC	FA)	GGBS	TPFA				
Control	12.50	–	–	–	6.00	0.48	37.5	1.0
Cement-FA	6.25	6.25	–	–	6.00	0.48	37.5	–
Cement-GGBS	6.25	–	6.25	–	6.00	0.48	37.5	1.5
Cement-TPFA	6.25	–	–	6.25	9.75	0.78	37.5	2.0

For mixing, the sand and binder were firstly dry mixed for 2 min. In next phase, a mixture was prepared owing 70 % of water and SP by continuing blending for 180 s. The remaining water was then poured to continue blending for further 300 s. The workability of mortars was determined through the flow table test.

The 50 mm cube steel moulds were used for making samples. Bi-layered cubes were shaped using fresh Mortar. Vibratory table was used to compact each layer. The demoulding was performed after 24 Hours. The curing of samples was carried out under 4 curing patterns, as named in Table 3. The durations for the curing were set to be 1 day, 2 days, 1 week, 4 weeks, 8 weeks, 3 months and 5 months. CS at early and delayed ages were considered in all evaluations because of the samples contained SCMs. The chemical and mechanical properties of the mixes at the delayed age of 3 months were expected to show no significant changes. Moreover, the information related to the pH value of CBMs at the later ages, is of prime importance to evaluate the durability.

**Table 3:** Detail of curing patterns.

Curing Pattern	Acronym
Room temperature curing in air	AC
Room temperature in Water	WC
Air curing at room temperature after hot water curing for 20 h at 60 °C	HAC
Water curing at room temperature after hot water curing for 20 h at 60 °C	HWC

### 2.3 Testing methodology

The flow of the mixtures was measured using flow table test. Every mixture was sustained with appropriate workability owing the flow of 21.5 ± 1 cm. The mentioned CS readings were yielded by averaging the findings of 3 samples. The CS test was conducted in accordance with the standard [35].

The pH was studied on the samples from inner parts of all the specimens. These samples were prepared using grinding machine [36]. The pH was measured using samples of 20 gm powder. Each sample of powder was mixed with distilled water (40 gm) aiming dilution of 1:2, as per the recommendations [37].

The stirring of the solution was carried out for 15 min. This solution was filtered using a 40-numbered filter paper, owing 110 mm. The average of 3 pH readings is reported for each mix. The pH measurement was performed using a digital pH meter and the samples were not stirred during pH measurement. Buffer solutions of pH 7.01 and 4.01 were used before each measurement in order to ensure calibration.

The TGA was performed to evaluate the content of Hydrated lime in mortars at the curing age of 2 days for WC & HWC samples. Similarly, for WC & HAC curing patterns, the tests were performed at the curing age of 4 weeks and 5 months. For TGA almost 100 mg samples were heated in a range of 30–1000 °C at 10 ^o^C/min heating rate. The Nitrogen flow rate was set to be 20 ml/min [[38], [39], [40]] In the light of earlier reports the estimation of Hydrated lime content was carried out in 300–550 °C range.

The produced samples WC and HWC owing curing period 2 days and WC and HWC curing period of 2 days and WC and HAC curing period of 4 weeks and 5 months respectively were tested using XRD. Major and minor phases present in mortars were determined using XRD data [[41], [42], [43]]. The findings from TGA on the Hydrated lime contents, were also endorsed by the diffraction patterns of the mixes at different curing ages. The specimens of all mortars were scanned using an X-ray diffractometer with a 2θ angle from 5° to 90°.

## 3 Results and discussion

### 3.1 Compressive strength development

The 1-day result of CS for the control, Cement-FA, Cement-GGBS and Cement-TPFA mortars are shown in Table 4. The standard deviation of 1-day CS of the control, Cement-FA, Cement-GGBS and Cement-TPFA mixes was 1.0, 0.8, 0.7 and 0.7; and the average standard deviation of 2-days CS was 1.9, 1.4, 2.3 and 1.0, respectively. The results showed that the CS of blended cement mortars at the age of 1 day reduced significantly due to the replacement of 50 % of OPC with FA, GGBS and TPFA. The CSs of the Cement-FA, Cement-GGBS and Cement-TPFA mixes were 68 %, 44 % and 81 % lower than those of the control mix, respectively.

**Table 4:** CS test results (MPa) at the age of 1 day.

Mix	CS (MPa)
Control	19.5
Cement-FA	6.1
Cement-GGBS	10.9
Cement-TPFA	3.6

**Fig. 1:**
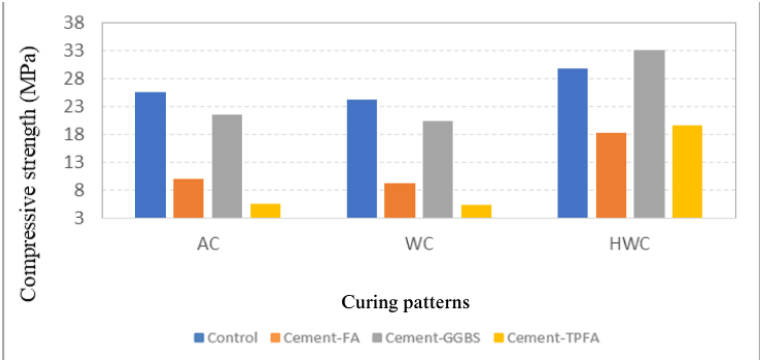
Curing pattern's effect on the CS upon 2 days curing.

The CSs of all mortars at 2-day age under all curing patterns are presented in Fig. 1. The results showed that CSs of all samples enhanced due to the curing method of HWC (for 20 h at 60 °C). As can be concluded from the results, HWC for 20 h at 60 °C can compensate for the reduction in CS because of the replacement of OPC with SCMs [44]. The results showed that under curing patterns AC & WC, the CS of Cement-GGBS mix was similar to that of the control mix. Strength of Cement-GGBS under the HWC curing pattern was 10 % more than that of the control mix. On the other hand, under WC and AC curing patterns, the CSs of the Cement-FA and Cement-TPFA mixes were approximately 62 % and 78 % less than those of the control mix, respectively. The CS of Cement-FA under HWC for 20 h at 60 °C significantly improved by up to 100 % relative to that under AC and WC curing patterns. The strength of Cement-TPFA showed a maximum increase of approximately 250 % under HWC for 20 h at 60 °C relative to that under AC and WC curing patterns. The average standard deviation of the CSs of the control, Cement-FA, Cement-GGBS and Cement-TPFA mixes at 7 and later ages was 2.10, 2.00, 2.39 and 1,89, respectively.

**Fig. 2:**
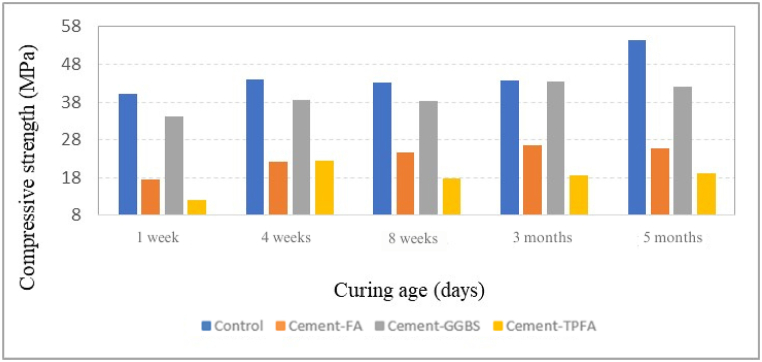
Curing age's effect on the CS of the AC samples.

**Fig. 3:**
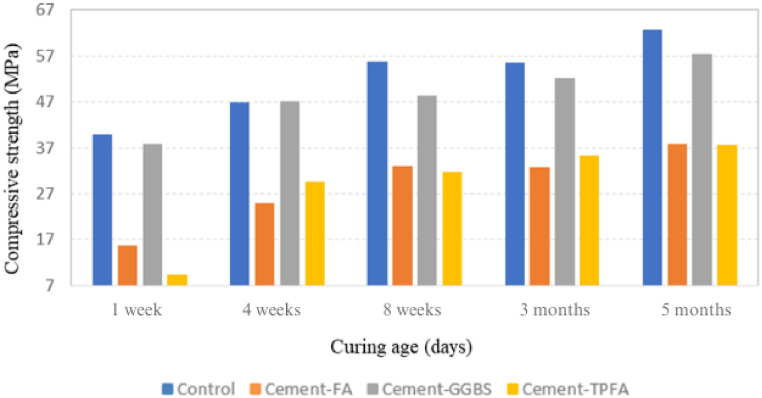
Curing age's effect on the CS of the WC samples.

**Fig. 4:**
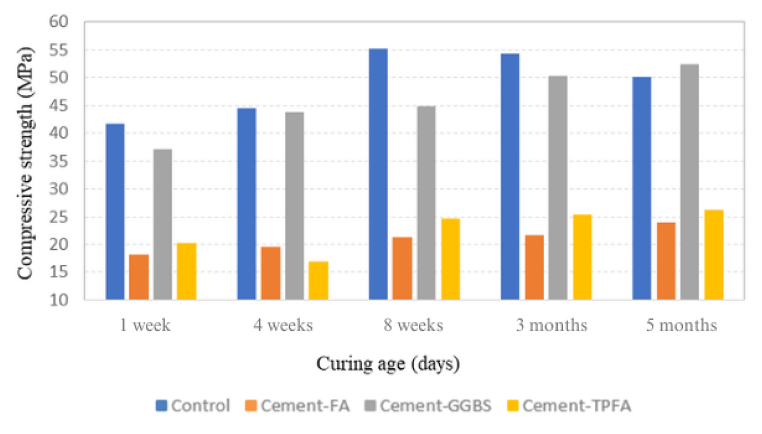
Curing age's effect on the CS of the HWC samples.

**Fig. 5:**
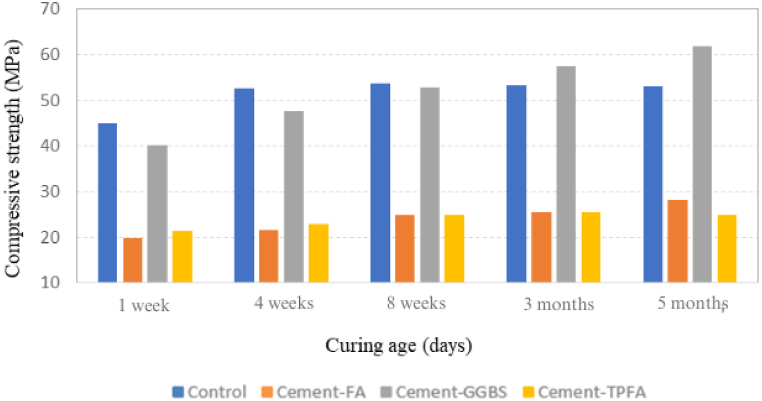
Curing age's effect on the CS of the HAC samples.

CS results of all mixes at 1 week, 4 weeks, 8 weeks, 3 months and 5 months under AC, WC, HWC and HAC curing patterns are provided in Fig. 2, Fig. 3, Fig. 4, Fig. 5, respectively. The findings obviously indicate that in comparison with the AC, WC and HWC curing patterns, the HAC curing pattern resulted in the highest CS of all mixes. The relative humidity and air temperature for AC samples were approximately 85 % and 28 °C ± 2 °C, respectively, whereas those for WC samples were 100 % and approximately 25 °C, respectively. The results indicated that under all curing patterns, the CS of Cement-GGBS was equivalent to that of the control mix.

Moreover, at the ages of 4 weeks–5 months, the CSs of Cement-FA and Cement-TPFA were nearly comparable and were approximately 50%–55 % less than those of the control sample under each curing pattern. The lower 4 weeks strength of Cement-FA mix compared to the control can be attributed to the slower rate of the PR. These findings were in agreement with the earlier reports. The research [45] investigates the effects of replacing a significant portion of Portland Cement with FA (typically around 50–60 %) in concrete mixtures. It reports that early-age strength is notably reduced in high-volume FA mixtures, and although there is a strength gain over time, it frequently remains lower than conventional concrete even at 3 months. The research [46] compares various FA content levels in concrete and their impact on CS and found that high levels of FA (over 40 %) generally lead to lower strengths, even after 3 months. This is attributed to slower PRs and limited calcium hydroxide availability from the lower cement content. High-volume FA mix requires more time to fully benefit from the strength-enhancing effects of the PR. This finding is consistent with previous research [47], which reflected that pastes owing a high volume of FA exhibited a slower reaction rate. In fact, in pastes with 45–55 % FA, more than 80 % of the FA did not react despite completion of 3 months curing period.

### 3.2 The pH behavior

The findings of pH testing results of the control, Cement-FA, Cement-GGBS and Cement-TPFA mortars under various curing patterns at the ages of 1 day, 2 days, 4 weeks and 5 months are presented in Table 5, Table 6, Table 7, respectively. The 1-day pH of all cement mortars in this work were in close agreement with the results of former studies showing that CBMs start their lives with high pH values of approximately 12.5–13.5 [3,48]. The results presented in Table 6 suggest that pH behaviour of the studied mortars and control samples decreased under accelerated curing for 20 h at 60 °C. However, the pH reduction exhibited by the Cement-TPFA mix was more pronounced than that exhibited by the Cement-FA and Cement-GGBS mortar mixes.

**Table 5:** The pH values of all cement mortars at 1 day age.

Mix	The pH value
Control	12.7
Cement-FA	12.5
Cement-GGBS	12.4
Cement-TPFA	12.3

**Table 6:** The pH values of all cement mortars under different curing patterns at 2 days age.

Mix	The pH value		
	AC	WC	HWC for 20 h at 60 °C
Control	12.5	12.5	12.3
Cement-FA	12.4	12.4	12.3
Cement-GGBS	12.3	12.4	12.2
Cement-TPFA	12.2	12.2	11.9

**Table 7:** The pH behavior of all Cement mortars under different curing patterns at4 weeks and 5 months.

Mix	Curing pattern	The pH value at	
		4 weeks	5 months
Control	WC	12.3	12.1
	AC	12.4	12.0
	HWC	12.3	12.1
	HAC	12.4	12.2
Cement-FA	WC	12.2	11.8
	AC	12.3	11.8
	HWC	12.3	11.9
	HAC	12.3	11.9
Cement-GGBS	WC	12.2	11.9
	AC	12.3	11.8
	HWC	12.3	11.9
	HAC	12.4	12.0
Cement-TPFA	WC	11.8	10.2
	AC	11.7	10.6
	HWC	11.8	10.4
	HAC	11.5	10.5

Table 7 shows that as described in a previous study [49], the pH behaviour of all the blended and control mortars exhibited a downward trend from 4 weeks till 5months. The reduction in pH was highly significant at the later age of 150-day in the case of the Cement-TPFA mortar under all curing patterns. The minimum and maximum reductions in the pH values of the Cement-TPFA mix were approximately 1.60 % and 13.60 %, respectively. Therefore, the pH values of CBMs should be considered, monitored and measured over the short and long terms. The results revealed that curing patterns had no significant effect on pH values of mortars. Using the Cement-TPFA mix as an example, the maximum pH difference due to WC, AC, HWC and HAC curing pattern was approximately 2.5 %. The standard deviation of the pH values of the control, Cement-FA, Cement-GGBS and Cement-TPFA mixes was 0.12, 0.12, 0.15 and 0.14, respectively.

All the results for pH in this work agreed with those in previous studies [28,50,51] describing that SCMs reduce high alkalinity, fill voids and harden the matrix, thus leading to a reduction in porosity and permeability. When a significant portion of cement is replaced with slow-reacting pozzolanic materials, there is a reduction in the formation of hydration products, particularly calcium silicate hydrate (C-S-H). This slower reaction at higher replacement levels is attributed to a decrease in the concrete's pH, which results from the reduced availability of calcium hydroxide (Ca(OH)₂). The solubility of amorphous silicates decrease as a consequence, leading to further slowdown of PR [52]. The obtained pH values were within the safe and essential range for the passivation of steel rebar film [53] and protection from carbonation and biodegradation [54] as well as chloride ingress [55]. However, reduction of pH of concrete even a small amount is of prime significance because the allowable limit for the synthesis of portlandite is 11.5–13 [[56], [57], [58]].

### 3.3 Variation in the quantity of hydrated lime

The TGA test was conducted on the 2-day-old WC and HWC mortars and 4 weeks and 5 months-old WC and HAC mortars. These curves provide the weight changes of the samples in relation to temperature changes and can help to evaluate the mass loss steps precisely.

**Fig. 6:**
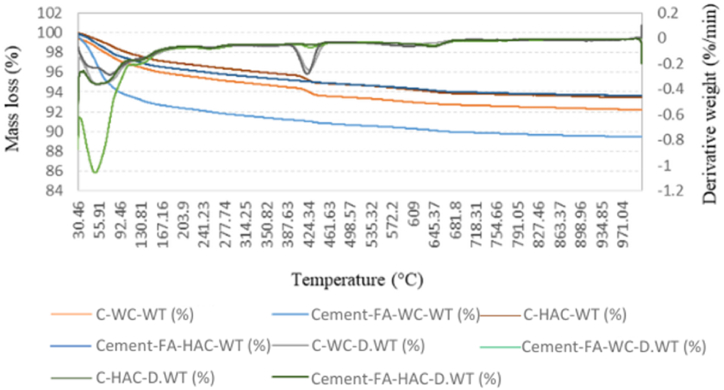
TGA curves of 4 weeks cured control and Cement-FA mortars under WC and HAC curing patterns.

**Fig. 7:**
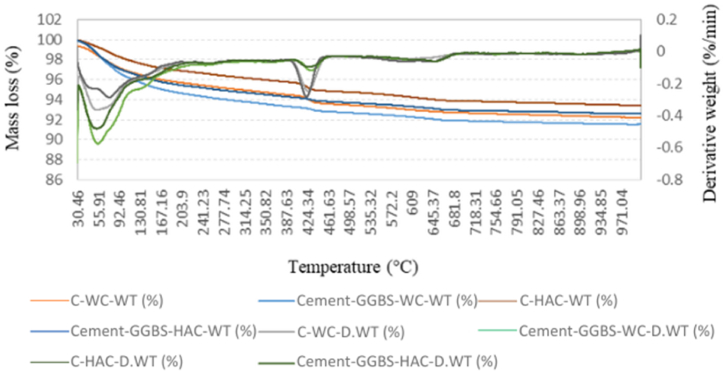
Control and Cement-GGBS mortars upon WC and HAC curing patterns.

**Fig. 8:**
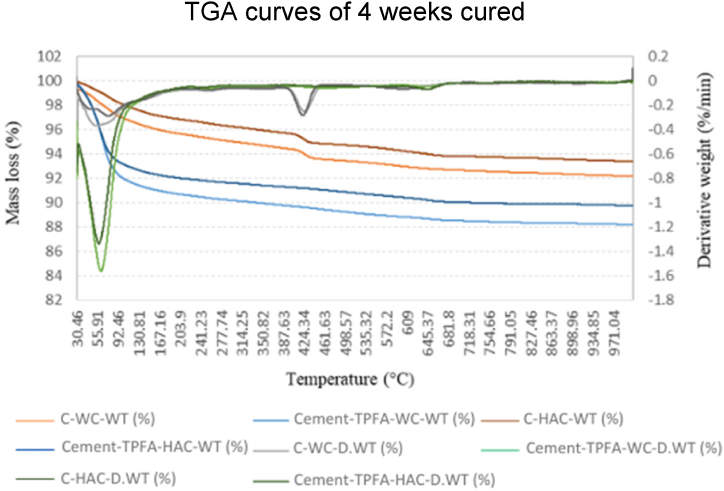
TGA curves of 4 weeks cured control and Cement-TPFA mortars upon WC and HAC curing patterns at 4 weeks.

Each TGA curve offered three main zones. In 1st zone, weight loss occurred within range of 30 °C-250 °C and revealed the evaporation of moisture contents, C–S–H and ettringite [58]. The 2nd zone from around 300 °C-550 °C showed the dehydration of Hydrated lime contents [38,40]. The 3rd mass loss appeared at about 600 °C and beyond symbolizing the whole decomposition of C–S–H and calcium carbonate (CaCO_3_) [59]. The TGA graphs of the control and Cement-FA, Cement-GGBS and -Cement-TPFA mixes cured under WC and HAC conditions at the ages of 4 weeks are shown in Fig. 6, Fig. 7, Fig. 8, respectively.

The TGA results for the Hydrated lime contents of the control, Cement-FA, Cement-GGBS and -Cement-TPFA blended cement mortars cured under WC, HWC and HAC conditions at ages of 2 days, 4 weeks and 5 months are summarised in Table 8. Results revealed that the Hydrated lime contents of the control mix exceeded those of all blended cement mortars and increased with time because of the higher amount of OPC in the control sample than being available in the blended mortars. Hydrated lime was produced via the OPC's hydration through the reaction of tricalcium silicate & dicalcium silicate [60].

**Table 8:** TGA results for the Hydrated lime contents of the control, FA, GGBS and TPFA blended cement mortars under WC, HWC and HAC curing.

Mix	Curing pattern	Hydrated lime contents from TGA (%)		
		2 days	4 weeks	5 months
Control	WC	1.468	1.754	2.609
	HWC	1.414	–	–
	HAC	–	1.688	2.196
Cement-FA	WC	1.163	1.423	1.572
	HWC	1.071	–	–
	HAC	–	1.075	1.604
Cement-GGBS	WC	1.360	1.252	1.223
	HWC	2.227	–	–
	HAC	–	2.046	1.242
Cement-TPFA	WC	1.433	1.338	1.165
	HWC	1.317	–	–
	HAC	–	0.997	0.958

The TGA findings discovered that the Hydrated lime contents of the Cement-FA mix increased over 2days−5 months. This conduct presented that the PR of FA was very slow even at the advanced age of 5 months. As can be observed from the outcomes, the filler effect of FA dominated the pozzolanic effect up to age of 5 months. These findings were in harmony with earlier findings displaying that the PR of FA may be enhanced at the later ages of 6–12 months [61,62]. Earlier researchers [63,64] described that the filler effect of FA is equally vital or even more than its pozzolanic effect. The filler effect of FA upsurges more than its pozzolanic effect with time, and the small particles of FA remain less reactive than those of OPC [65]. Therefore, this behaviour of FA may result in the action of several unreacted FA particles as space fillers.

The TGA findings directed that the PR of GGBS consumed Hydrated lime over 2 days to 5 months. The reduced Hydrated lime contents caused dilution due to the low cement content and PR of the Cement-GGBS mortar mix. The self-hydration of GGBS and OPC produces Hydrated lime [66]. Total Hydrated lime content in pore solution is dependent on its formation and consumption rates. Previous studies [67,68] have reported that the Hydrated lime content depends on the self-hydration rate of GGBS and cement, the PR of GGBS and the replacement level of GGBS with OPC.

The TGA results illustrated that in the case of the Cement-TPFA mix, the Hydrated lime content decreased with the prolongation of curing time because of the ingestion of Hydrated lime during the PR of -TPFA to formulate C–S–H gel and the dilution of cement through cement hydration. The Hydrated lime mass loss of Cement-TPFA mix at 4 weeks of WC curing was approximately 6.63 % and increased to approximately 12.93 % at 5 months under WC conditions. This result showed that PR of TPFA was more dominant at the later age of 5 months.

### 3.4 XRD

**Fig. 9:**
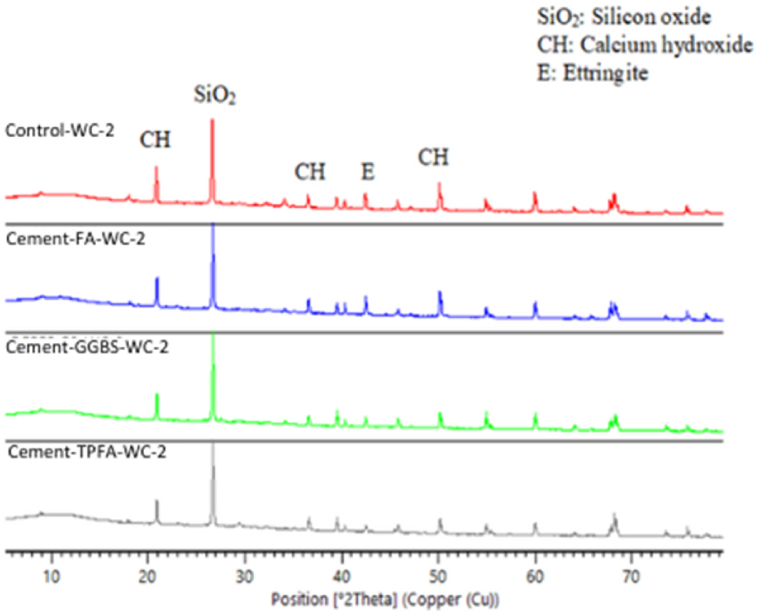
XRD patterns of control, Cement-FA, Cement-GGBS and Cement-TPFA mortars at 2 days curing in WC curing pattern.

**Fig. 10:**
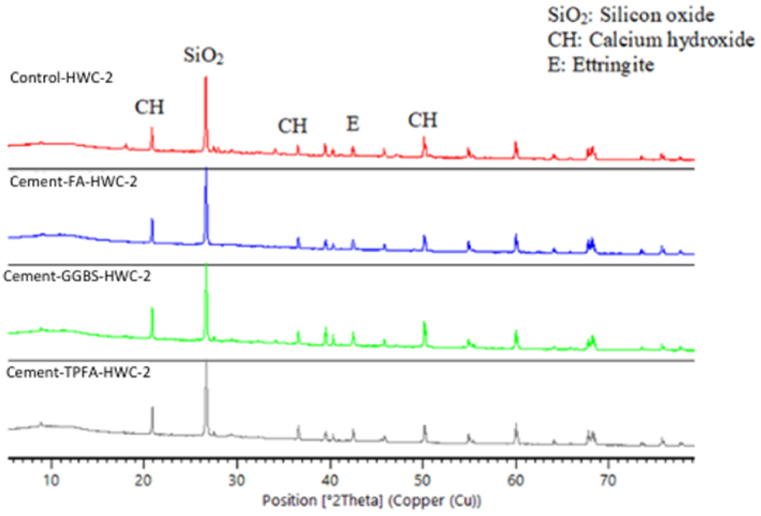
XRD patterns of control, Cement-FA, Cement-GGBS and Cement-TPFA mortars at 2 days curing in HWC curing pattern.

**Fig. 11:**
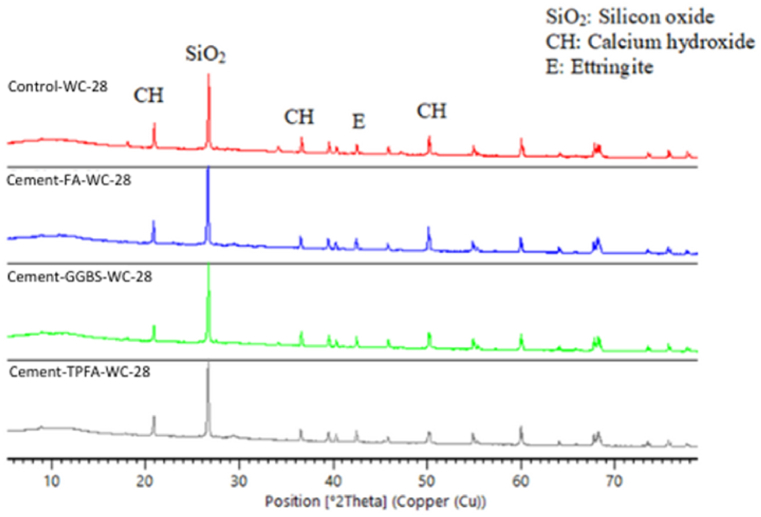
XRD patterns of control, Cement-FA, Cement-GGBS and T-Cement-TPFA mortars cured for 4 weeks under WC curing pattern.

**Fig. 12:**
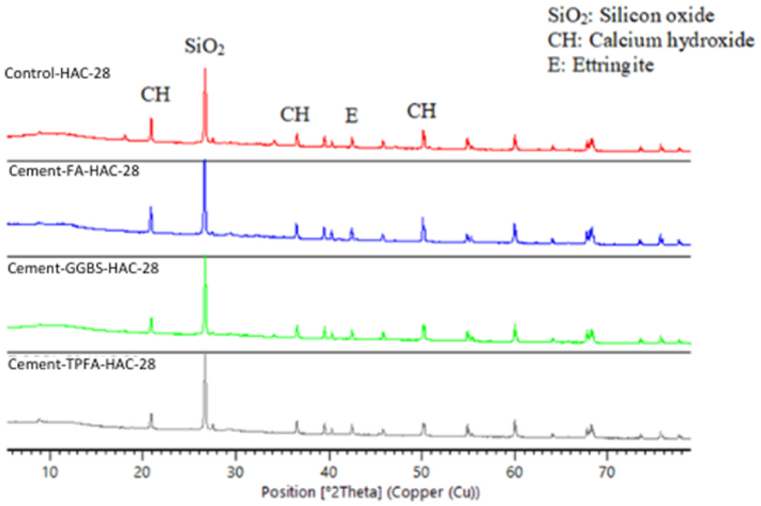
XRD patterns of control, Cement-FA, Cement-GGBS and T-Cement-TPFA mortars cured for 4 weeks under HAC curing pattern.

**Fig. 13:**
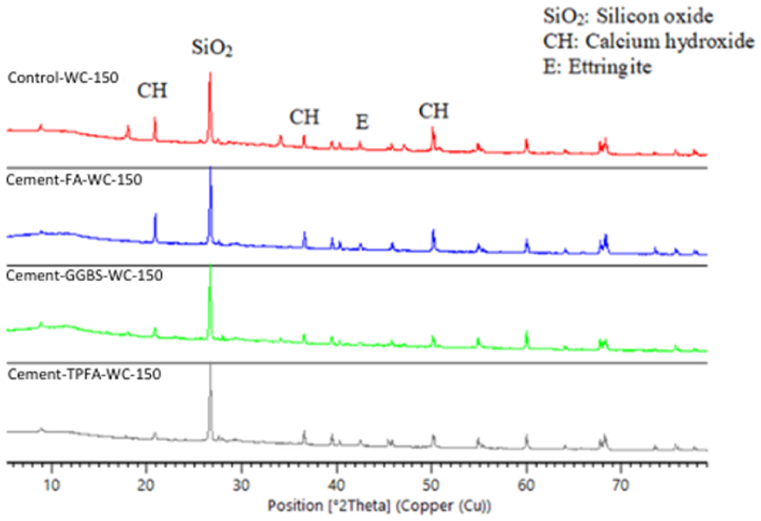
XRD patterns of control, Cement-FA, Cement-GGBS and T-Cement-TPFA mortars cured for 5 moths under WC curing pattern.

**Fig. 14:**
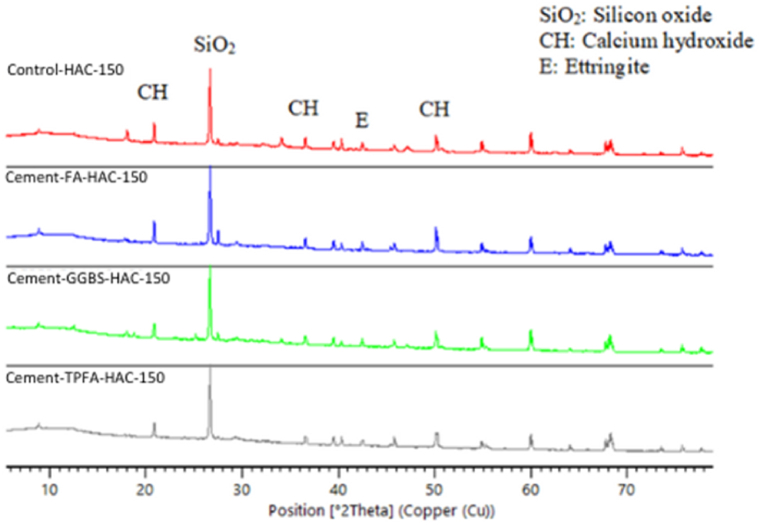
XRD patterns of control, Cement-FA, Cement-GGBS and -Cement-TPFA mortars cured for 5 months under HAC curing pattern.

The XRD patterns of the hydrated mortars of the control, Cement-FA, Cement-GGBS and Cement-TPFA under WC, HWC and HAC curing patterns at the ages of 2 days, 4 weeks and 5 months are shown in Fig. 9, Fig. 10, Fig. 11, Fig. 12, Fig. 13, Fig. 14, respectively. The XRD patterns clearly illustrated that in all types of mortars, silicon oxide (SiO_2_) was detected in the form of a major peak due to the presence of sand in the mixes [[69], [70], [71]]. Notably, this SiO_2_ was not a hydration product but was instead due to the presence of a large quantity of sand in all the mortar mixes.

The XRD patterns reflected the formation of large quantities of Hydrated lime in the mixes, particularly in the control and Cement-FA mixes. These findings were in harmony with the TGA findings for Hydrated lime in all mortar mixes. Moreover, ettringite was formed at the initial stage of hydration. At later ages, no significant ettringite peaks can be found in all mortars. As can be seen from the XRD patterns, the amount of Hydrated lime decreased with time due to the PR of GGBS and TPFA in their respective mixes. In the case of the Cement-FA mix, the peaks corresponding to Hydrated lime reflected slow PR and filler effect of FA with the passage of time. Overall, all the XRD patterns of the pure and blended cement mortars validated the TGA results for the changes in Hydrated lime content with curing age.

As can be concluded from the TGA and XRD results, FA exerted a filler effect up to the age of5 months. Therefore, Hydrated lime could not be consumed due to the existence of FA in the blended cement mortars. However, under all curing patterns, the pH values of the Cement-FA blended cement mortars were lower than those of the control mortar and decreased with time. Therefore, the pH value wasn't only reliant on the Hydrated lime content resulted from cement hydration. This result was in line with the findings of former studies [40,58,72] showing that the total pH value of CBMs is dependent on the quantities of Hydrated lime, KOH and NaOH. The results of this study revealed the absence of a direct relationship between Hydrated lime contents and pH in mortar mixes. Therefore, the other parameters influencing the pH of CBMs should be considered, and further studies are required.

## 4 Conclusions

FA, GGBS and TPFA were employed to replace 50 % of OPC in cement mortars and consequent effect on the pH behaviour was evaluated. The mortars were cured under different conditions, such as standard water curing, accelerated curing (using hot water) and air drying. The following conclusions were drawn from the test results.

1. The pH of the control and all blended cement mortars slightly reduced under HWC at 60 °C for 20 h after demoulding. However, the pH reduction in Cement-TPFA was more than the other two blended cement mortars and was approximately 2.5 %.
2. Irrespective of the curing method, pH values of the blended cement mortars decreased over time. The pH measurement showed that the pH of the blended cement mortars containing GGBS or FA remained within the safe range (more than 11.5) for up to 5 months. However, the mortars containing TPFA showed significant reductions in pH from 11.5 to 11.8 to 10.2–10.6. Therefore, given that durability is a major concern for concrete structures, the usage of high volumes of TPFA in reinforced concrete is not recommended.
3. Although some previous studies have highlighted that the consumption of Hydrated lime in CBMs accounts for the reduction in the pH of blended CBMs, this study showed that no direct relationship existed between the amount of Hydrated lime from cement hydration process and pH behavior of the blended cement mortar.

## CRediT authorship contribution statement

**Sumra Yousuf:** Conceptualization. **Muhammad Rizwan:** Formal analysis. **Belal Alsubari:** Supervision. **Mustabshirha Gul:** Investigation. **Muhammad Mahmood Ali:** Funding acquisition. **Muhammad Nasir Bashir:** Funding acquisition. **Abid Latif:** Resources.

## Data and code availability statement

The data will be made available on request.

## Compliance with ethical standards

The authors have reviewed Ethics in Publishing as well as Heliyon's Ethics and Editorial Policies for this research.

## Declaration of competing interest

All the authors declare no conflict of interest.
